# Design and Performance Analysis of a Hybrid Flexible Pressure Sensor with Wide Linearity and High Sensitivity

**DOI:** 10.3390/s26010238

**Published:** 2025-12-30

**Authors:** Qinghua Zhang, Zhenxing Liu, Jianbo Wu, Ping Sun, Hanwen Zhang

**Affiliations:** 1 Artificial Intelligence School, Wuchang University of Technology, Wuhan 430223, China; sqmha@126.com; 2 College of Optoelectronic Engineering, Chengdu University of Information Technology, Chengdu 610225, China; 13290971072@163.com (Z.L.);

**Keywords:** flexible pressure sensors, gradient non-uniform porous structure, PVC/CNT composite materials, wide linear range, orthogonal experiment optimization

## Abstract

**Highlights:**

A flexible pressure sensor with a gradient non-uniform porous structure was developed, achieving synergistic optimization of high sensitivity (5.57 kPa^−1^) and wide linear range (0–120 kPa, R^2^ > 0.99) via PVC/CNT composite and bimodal sugar template design. The sensor exhibits excellent dynamic performance (80 ms response time, 50 ms recovery time) and cyclic stability (>500 cycles), providing a reliable solution for high-precision pressure detection in complex scenarios.

**What are the main findings?**

- A gradient non-uniform porous structure design is proposed, using PVC/CNT composite slurry as the sensing layer and a bimodal sugar template (50–75 μm + 125–150 μm particles) to construct pores. Through orthogonal experiment optimization, the optimal parameters are determined as 3 wt% CNT concentration and a 10:3 mass ratio of PVC/CNT to sugar template.
- The optimized sensor demonstrates outstanding comprehensive performance: high linearity (R^2^ > 0.99) and high sensitivity (5.57 kPa^−1^) over 0–120 kPa, millisecond-level dynamic response, cyclic stability exceeding 500 cycles, and signal fluctuation less than 5%, attributed to the synergistic deformation mechanism of large and small pores.

**What are the implications of the main findings?**

- This study breaks through the performance trade-off between sensitivity and wide linear range in traditional flexible piezoresistive pressure sensors, offering a new technical approach for performance optimization of such sensors.
- The sensor shows significant application potential in wearable health monitoring, electronic skin, and other cutting-edge fields, while providing a theoretical and experimental basis for future research on precise pore regulation and signal decoupling integration.

**Abstract:**

This study presents a wide-linear-range flexible pressure sensor based on a gradient non-uniform porous structure. Through co-optimization of material composition and structural parameters, the sensor integrates high sensitivity, a broad linear response range, and excellent stability. The sensing layer is fabricated using a PVC/CNT composite slurry, with interdigital silver electrodes screen-printed on a PET substrate. A porous architecture is constructed via solution blending and a template method. Innovatively, orthogonal experiments were employed to optimize the conductive filler concentration and porosity. A mixed sugar template comprising particles of 50–75 μm and 125–150 μm was introduced to form a gradient non-uniform porous structure, effectively expanding the linear response range. Experimental results demonstrate that the sensor exhibits outstanding linearity (R^2^ > 0.99) and high sensitivity (5.57 kPa^−1^) over a broad pressure range of 0–120 kPa. It also shows a dynamic response speed of 50 ms, cyclic stability exceeding 500 cycles, and signal fluctuation of less than 5%. Scanning electron microscopy (SEM) analysis reveals the synergistic mechanism of the non-uniform pores, confirming the effectiveness of this design in reconciling the trade-off between sensitivity and linear range. This study offers new insights into the performance optimization of flexible pressure sensors and demonstrates significant potential for applications in health monitoring and electronic skin (E-skin).

## 1. Introduction

Flexible piezoresistive pressure sensors, as the core components of wearable electronics and intelligent monitoring systems, have achieved breakthrough progress in the fields of materials science and microstructure engineering in recent years [1,2,3]. Compared with conventional rigid sensors, which are limited by issues such as volume, weight, and mechanical adaptability, this technology, through the collaborative design of flexible substrates and conductive materials, not only achieves lightweight and high-sensitivity devices but also exhibits excellent conformability for monitoring human movement and physiological signals [4,5]. However, existing studies generally face a performance trade-off between sensitivity (S) and linear detection range (LR), whose essence stems from the nonlinear coupling effect between the deformation mechanism of the piezoresistive layer and the evolution law of the conductive network [6,7,8,9]. This contradiction is particularly prominent in complex pressure scenarios. Highly sensitive sensors typically exhibit conductive network saturation in the low-pressure region (<10 kPa), while wide-linearity sensors struggle to achieve accurate detection of tiny physiological signals (<1 kPa) simultaneously [10,11]. Addressing the above challenges, this study focuses on optimization strategies for sensing performance, systematically summarizes the latest research progress of scholars in microstructure topology regulation and multi-level conductive network construction, aims to reveal the synergistic mechanism between sensitivity enhancement and linear-range expansion, and provides a theoretical basis and technical approach for developing high-performance flexible sensing systems adapted to complex scenarios.

In the direction of sensitivity optimization, the research focus is on the coordinated regulation of microstructure engineering and the dynamic reconstruction of conductive networks [12,13]. Surface microstructure design significantly improves the incremental rate of contact area per unit pressure through the stress concentration effect; in this context, conical arrays have attracted considerable attention due to their optimal stress transfer efficiency [14,15,16]. Cheng et al. [17] developed a conical PDMS/graphene composite sensor that achieves a sensitivity of 0.122 kPa^−1^ in the range of 0–5 kPa through an apex-localized strain amplification mechanism. Its performance enhancement stems from the gradient contact area expansion characteristic of the conical array (ΔA/A_0_ ≈ 3.2P0.5). Bionic microstructure design further expands the dimensions of performance optimization. The Wang group [18] constructed a Au-PDMS multilayer sensor mimicking the fractal structure of reed leaves, and utilized the stress-focusing effect of the groove structure to increase the sensitivity to 2.54 kPa^−1^ (0–107 kPa), confirming the unique advantages of bionic topology in wide-range sensing. Porous structure design breaks through the sensitivity bottleneck via 3D conductive network reconstruction. Cui et al. [19] employed a multi-level pore structure constructed from CNT sponge to achieve an ultra-high sensitivity of 809 kPa^−1^ in the range of <10 kPa. The mechanism lies in the fact that pore compression triggers elastic buckling of microfibers (buckling strain ε_c ≈ 15%), which promotes the exponential growth of contact points (N_c ∝e0.12P). Notably, such structures can achieve a balance between sensitivity and mechanical stability by regulating the porosity gradient (30–80%) and feature size (10–500 μm), with a typical cycle life of over 4000 cycles (ΔR/R_0_ decay < 8%).

Research on linear-range expansion is dedicated to addressing the coupling challenge of nonlinear compression in piezoresistive materials and conductive network saturation, whose core lies in establishing a linear stress-conductance transfer mechanism. Hierarchical porous architectures enable stage-specific regulation of pressure transmission through gradient modulus design: Zhu et al. [20] developed a bionic gradient porous sensor that optimizes pore distribution via finite element analysis (porosity gradient: 30–70%) and maintains a linearity of R^2^ > 0.99 in the range of 0–500 kPa. The mechanism lies in the preferential collapse of macropores (200–500 μm) triggering conductive network reconstruction, while the gradual compression of micropores (<50 μm) delays pathway saturation. The inverse lattice porous structure designed by the Bang group [21] maintains R^2^ = 0.998 over an ultra-wide range of 0.03–1630 kPa through a lattice contact phase transition mechanism. Its mathematical essence is the conversion of the nonlinear growth of MWCNT contact area (ΔA ∝ P1.5) into a linear output. A breakthrough advance stems from the multi-physical field coupling strategy: Huang et al. [22] constructed modulus-gradient elastomers (0.1–5 MPa) via magnetic field-directed alignment, achieving a linear response with R^2^ = 0.999 in the range of 80 Pa−220 kPa. The sensor relies on matrix flexibility (E ≈ 85 kPa) in the low-pressure region and suppresses structural collapse via magneto-induced hardening (ΔE ≈ 470%) in the high-pressure region. These findings indicate that a synergistic enhancement model for S-LR (K = ΔS/ΔLR ≈ 0.32) can be established through pore gradient design and dynamic contact mechanism modulation, providing a theoretical framework for wide-domain linearized sensing.

In this contribution, addressing the shortcomings of flexible piezoresistive pressure sensors in terms of sensitivity and linearity, appropriate sensing active materials and substrate materials were selected, and devices were fabricated via simple mixing, curing, and washing methods. By adjusting the composition ratio of the conductive composite, using sugar particles (SPs) as a sacrificial template, and regulating the particle size of SPs to control the gradient of the porous structure, a composite conductive material with a wide-linearity non-uniform porous structure was designed. This study designs the fabrication process and procedure of the material, characterizes its morphology, and clarifies the conditions and mechanisms for the sensor to achieve high sensitivity and a wide linear sensing range. This research provides an effective strategy for flexible piezoresistive pressure sensors to maintain good linearity over a wide pressure detection range, and simultaneously holds considerable practical significance for the design and construction of high-performance flexible pressure sensors at the microscopic level.

## 2. Materials and Methods

### 2.1. The Preparation of Composite Conductive Sensing Layer

PVC is a common polymer with a wide range of applications, and is favored for its unique properties and economic advantages. Firstly, PVC exhibits excellent chemical stability and corrosion resistance, enabling long-term stable operation even in humid, acid–alkaline, or organic solvent environments. Secondly, its low raw material cost and mature processing technologies have made it one of the most industrialized polymers. Owing to its thermoplastic properties, PVC can be rapidly formed via processes such as hot pressing and injection molding, significantly reducing processing difficulty and production costs. Compared with silicone materials such as PDMS and Ecoflex, PVC is more suitable for mass production, providing a cost advantage for products like flexible sensors. Furthermore, medical-grade PVC has undergone long-term practical verification, possesses excellent biocompatibility, and has been widely used in medical devices such as infusion tubes and blood bags. This versatility not only meets the high-efficiency requirements of industrial production but also ensures the long-term reliability of products in various harsh environments.

Carbon nanotubes (CNTs) are used as conductive fillers. Their high electrical conductivity, low filling content, excellent mechanical flexibility, sensitive deformation response, and good composite compatibility with elastic matrices enable the sensor to form a continuous conductive network at a low filling ratio. This not only ensures the stability of the device during repeated bending and stretching but also significantly lowers the resistance change threshold, achieving high sensitivity and fast response. Additionally, during sensor preparation, via the solution-blending process, PVC can uniformly disperse CNTs (serving as conductive fillers). Moreover, the high viscosity of PVC can inhibit filler sedimentation during the curing process, ensuring the uniformity of the conductive network and achieving a favorable piezoresistive effect.

The selection of materials usually requires comprehensive consideration of material properties and cost. In this study, polyvinyl chloride (PVC) was used as the matrix material of the sensing layer, and carbon nanotubes (CNTs) were used as the conductive filler. PVC (AR, 99% purity) was obtained from Jaboli Chemical Group Co., Ltd. (Jiangmen, China). MWCNTs (95% purity, length less than 10 μm, diameter 10–30 nm) were used as CNT conductive material and received from Shenzhen Hongd Evolution Technology Ltd. (Shenzhen, China). Conductive silver paste was purchased from Hunan Guoyin New Material Co., Ltd. (Changsha, China). White sugar (SP) powder was obtained from Shanghai Wenkua Food Co., Ltd. (Shanghai, China).

**Figure 1:**
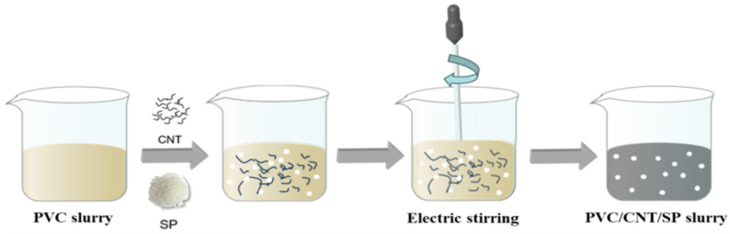
PVC/CNT/SP composite functional paste preparation process.

**Figure 2:**
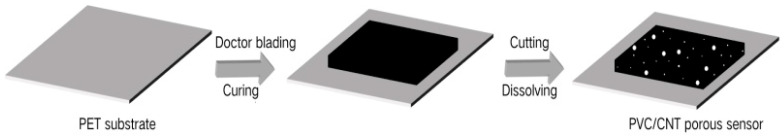
Flow of porous sensing layer preparation.

A simple solution-blending method was adopted herein (specific steps are shown in Figure 1): CNT raw material, PVC raw material, and white sugar (SP) powder were mixed to prepare the PVC/CNT/SP functional slurry. The preparation process of the sensing layer is shown in Figure 2: A doctor blade with a gap of 1 mm was used for blade coating, and the mixed slurry was coated on the surface of a PET flexible substrate to form a conductive film with a thickness of 1 mm. Subsequently, the coated samples were placed in a constant-temperature drying oven for curing at 60 °C for 3 h, ensuring that the film was completely dried and formed a stable structure. After curing and drying, the film was cut into 1 cm × 1 cm samples, immersed in deionized water, and magnetically stirred at 25 °C and 200 rpm for 6 h to thoroughly dissolve the white sugar template and form a porous structure. Finally, the samples were taken out, with residual surface moisture removed by cold-air blowing, and then dried in a vacuum-drying oven at 60 °C for 10 min to complete the preparation of the porous sensing layer.

### 2.2. Preparation of Electrode Layer

Silver is one of the most conductive materials among metals; therefore, the silver in silver paste exhibits excellent electrical conductivity. When used as an electrode material, it enables the electrode layer to transmit signals quickly and efficiently while reducing the overall resistance of the device. Interdigitated electrodes, through their interlocking finger-like structure, significantly expand the effective contact area between the electrodes and the sensing layer. They also facilitate integration into miniature and thin devices, meeting the miniaturization and thinness requirements of flexible electronic devices. Meanwhile, silver paste can directly form interdigitated structures on flexible substrates via printing processes. This method is easy to operate, low-cost, and conducive to large-scale production. Furthermore, the excellent fluidity and fillability of silver paste help form a continuous and uniform film on the substrate surface, thereby improving the adhesion between the electrodes and the substrate and reducing interface defects. Additionally, after combining with flexible polymers, silver paste maintains electrical stability during repeated bending, stretching, and twisting, resists cracking, and ensures stable signal transmission during long-term device operation.

**Figure 3:**
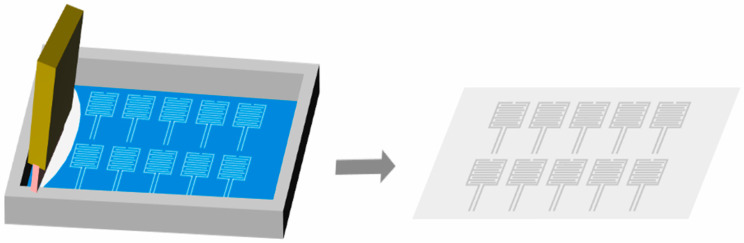
Electrode layer preparation process.

In summary, this study employs conductive silver paste as the electrode material and fabricates flexible interdigitated electrodes on flexible substrates via a simple screen-printing process. The experimental setup and process flow for preparing the electrode layer are shown in Figure 3: A PET flexible substrate is fixed on the stage of a screen-printing machine, and alignment marks are used to accurately align the interdigitated electrode template (finger width: 12 mm; finger spacing: 0.3 mm; overall size: 50 mm × 15 mm) with the substrate surface; subsequently, conductive silver paste is uniformly applied onto the polyester screen (mesh count: 250; equivalent aperture diameter: 58 μm), and a polyurethane squeegee is controlled to form a 45° angle with the screen. The paste is scraped unidirectionally at a constant pressure and uniform speed to ensure complete filling of the template pattern with the silver paste; finally, after removing the screen template, the PET substrate printed with interdigitated electrodes is transferred to a circulating-air oven and cured at 60 °C for 2 h to fully form the conductive path of the silver paste.

### 2.3. Packaging of Pressure Sensors with Porous Structure

**Figure 4:**
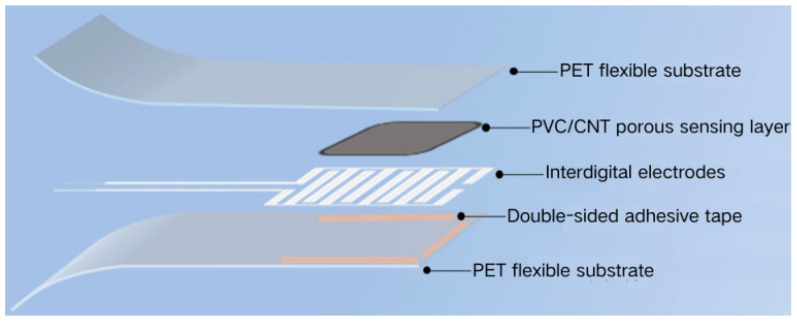
Porous pressure sensor structure schematic diagram.

The complete structure of the sensor is shown in Figure 4. The sensor assembly follows a bottom-up sequence: First, the silver paste interdigitated electrodes printed on the PET substrate serve as the complete electrode layer, which is placed at the bottom; subsequently, double-sided adhesive tape is applied to the surface of the electrode layer. This tape acts as an adhesive layer to fix the porous sensing layer (supported on another PET substrate). Finally, the porous PVC/CNT sensing film is placed on the interdigitated electrodes, ensuring uniform adhesion, followed by covering it with a top PET film substrate. The entire structure is bonded using double-sided adhesive tape. The top and bottom PET films function as insulating layers to seal the entire device, ultimately forming the flexible pressure sensor by an encapsulation machine.

### 2.4. Test Platform

To comprehensively evaluate the comprehensive performance of the fabricated wide-linearity flexible pressure sensors, this chapter conducts systematic testing and analysis of their key performance parameters, specifically including sensitivity, pressure gradient response characteristics, dynamic response speed, cyclic stability, and constant-pressure stability. Through experimental testing, this study not only verifies the effectiveness of the gradient non-uniform porous structure design in improving the sensor’s linear range and sensitivity but also reveals the sensor’s reliability and durability in complex application scenarios. These test results not only provide data support for the sensor’s performance optimization but also lay a theoretical foundation for the selection and adaptation of its practical application scenarios.

**Figure 5:**
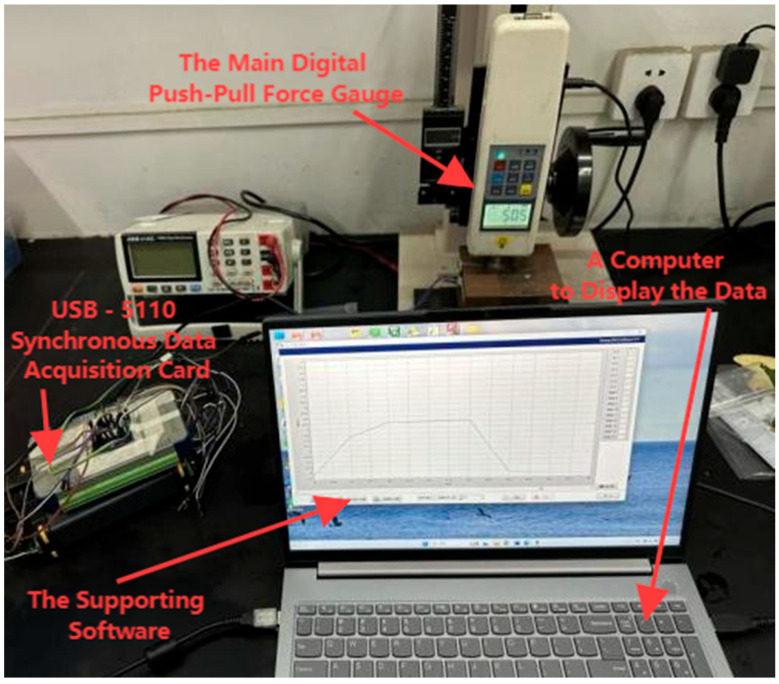
Testbed display.

To investigate the sensing characteristics of flexible pressure sensors based on PVC/CNT porous structures, this paper independently designed and built a dedicated pressure-sensing test platform to conduct a series of performance tests on the sensors. The physical composition of the test platform is shown in Figure 5, which mainly includes a digital display push–pull force gauge, a USB-5110 synchronous data acquisition card (Smacq) with supporting software, and one computer for data display. Among these components, the USB-5110 data acquisition card is connected to an external Wheatstone full-bridge circuit for signal acquisition. The calibrated balance resistor of the Wheatstone bridge and the sensor form a 1/4-bridge (quarter-bridge) configuration, which is used to capture the current variation values of the sensor in real time. Meanwhile, the data acquisition card transmits the collected data to the computer, and the supporting software completes data recording; the computer is responsible for saving and subsequent processing of the collected data.

## 3. Innovative and Optimized Design of Wide-Linearity Flexible Pressure Sensors

A wide linear response is a key characteristic for flexible pressure sensors to achieve high-precision detection in complex application scenarios (e.g., human motion monitoring, electronic skin). In this section, based on the design concept of synergistic optimization of the material system and porous structure, the orthogonal experimental method is employed to systematically investigate the influence of conductive filler concentration, porous structure type, and its gradient distribution on the sensor’s linear response range. Through characterization analysis and mechanism elaboration, a design scheme for a flexible pressure sensor with wide-linearity characteristics is proposed.

### 3.1. Design Strategy for Wide-Linearity Flexible Pressure Sensors

First, starting from the basic properties of the material, the balance relationship between the electrical conductivity, mechanical flexibility, and pressure sensitivity of the composite was investigated by adjusting the concentration of carbon nanotubes (CNTs), the conductive filler. The gradient concentration comparison method was used in the experiments to screen out the optimal concentration threshold that not only ensures the connectivity of the conductive network but also avoids material embrittlement caused by excessive fillers, laying a material foundation for subsequent structural design.

Carbon nanotubes (CNTs) of 5 g, 4 g, 3 g, 2 g, and 1 g were, respectively, blended with 100 g of polyvinyl chloride (PVC) raw material, followed by magnetic stirring at 400 rpm for 2 h using a precision force-enhanced electric stirrer until a homogeneous mixed system was formed. To screen out the optimal ratio of CNTs, a total of five types of PVC/CNT functional slurries with CNT mass fractions of 1 wt%, 2 wt%, 3 wt%, 4 wt%, and 5 wt% were prepared in this experiment.

For the PVC/CNT films with 2 wt%, 3 wt%, 4 wt%, and 5 wt% CNT contents (the CNTs used in the experiment are multi-walled carbon nanotubes, i.e., MWCNTs), their resistance values are 16.8 MΩ, 2.83 MΩ, 181.4 KΩ, and 4.61 KΩ, respectively. In contrast, the resistance of the PVC/CNT film with 1 wt% CNTs is too high, exceeding the maximum detection range of the digital multimeter, and exhibits approximately insulating properties.

**Figure 6:**
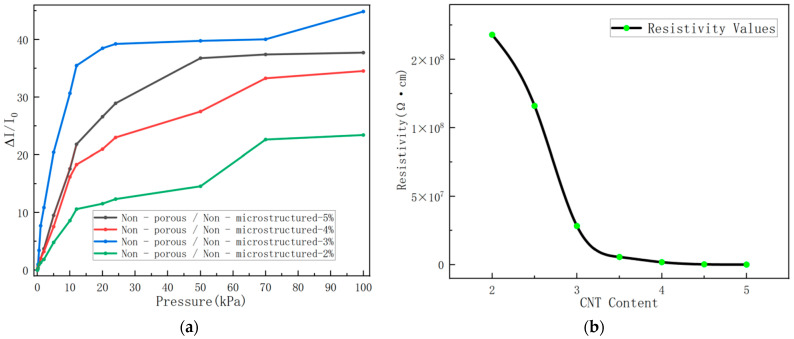
(**a**) Relative current change curves versus applied pressure for sensors based on PVC/CNT films with different CNTs. (**b**) Resistivity curves of conductive polymer composites with CNT content.

The concentration of carbon nanotubes (CNTs) directly affects the integrity of the conductive network. As shown in Figure 6a, the experimental results indicate that when the polyvinyl chloride/carbon nanotube (PVC/CNT) composite containing 3 wt% CNTs is used as the sensing layer, the sensor exhibits stable and favorable response, as well as the optimal sensitivity in the pressure range of 0–24 kPa. For the insulating PVC matrix, only when the CNT content exceeds the percolation threshold can the CNTs contact each other or be sufficiently close to form a continuous conductive network within the matrix. Specifically, for 1 wt% CNTs, the CNTs fail to form an adequate number of conductive paths, resulting in excessively high overall resistance—undetectable even by a digital multimeter—exhibiting “approximately non-conductive” behavior. For 2 wt% CNTs, although a certain number of CNT conductive paths are formed, the network is far from optimal, and the resistance remains relatively high (16.8 MΩ). As shown in Figure 6b, for 3 wt% CNTs, the CNT content is just around the “percolation threshold,” forming a relatively reasonable conductive network with a resistance value (2.83 MΩ) in an appropriate range. Under external force, the disconnection or contact state change of the CNT network is more pronounced, enabling a larger relative current change (ΔI/I_0_) and thus achieving higher sensitivity and better linearity. For 4 wt% and 5 wt% CNTs, the CNT content increases significantly, leading to a substantial increase in conductive paths and a reduction in resistance to the kilohm (KΩ) order of magnitude. Although the overall electrical conductivity is improved, excessive CNTs reduce the relative current change rate (ΔI/I_0_) of the material, thereby impairing the sensing sensitivity. In summary, the 3 wt% CNT concentration balances electrical conductivity and mechanical properties within the 0–24 kPa pressure range, so subsequent experiments were conducted using this CNT content for further investigation.

### 3.2. Optimization Study of the Porous Structure by Sacrificing the Mass Ratio of White Sugar Templates

Based on the 3 wt% CNT concentration selected in the previous subsection, porous structures were prepared via the sacrificial template method, using screened white sugar particles as the sacrificial template. Among the materials used, white sugar is a common raw material that offers low cost, large-scale availability, and no requirement for complex chemical treatment. It can be directly mixed with the PVC/CNT slurry, making the entire preparation process simple, non-toxic, and environmentally friendly. By applying different pressures to test the sensor’s electrical signal response, this study elucidated the correlation between porous distribution density and the sensor’s sensitivity and linearity, and clarified the porous structure characteristics required for a high linear response.

The 3 wt% PVC/CNT composite slurry and white sugar powder (aperture < 75 μm) were mixed at different mass ratios of 10:2, 10:3, 10:4, and 10:5, respectively. Each group was magnetically stirred at 400 rpm for 2 h using a force-enhanced electric stirrer until the white sugar powder and composite slurry were uniformly mixed, completing the slurry preparation. Subsequently, flexible pressure sensors were fabricated following the experimental procedures described before, yielding four groups of samples with different porous structures.

**Figure 7:**
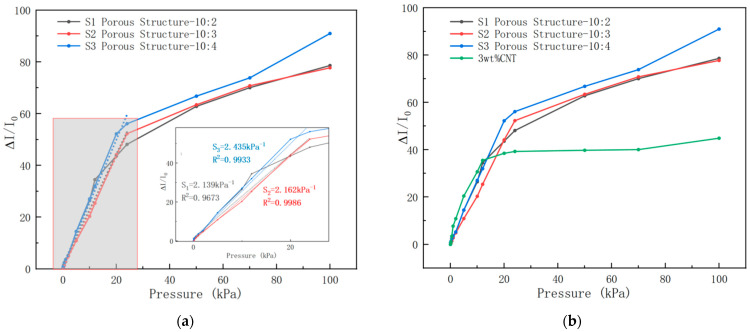
(**a**) Test results of porous structure samples with different mass ratios of 3 wt% CNT. (**b**) Comparison of porous structure samples with different mass ratios of 3 wt% CNT with non-porous-structure samples.

According to the test results in Figure 7a, within the pressure range of 0–24 kPa, the sensitivity and coefficient of determination (R^2^) of Porous Structure PVC/CNT/SP 10:2 are 2.139 kPa^−1^ and 0.9672, respectively; those of Porous Structure PVC/CNT/SP 10:3 are 2.162 kPa^−1^ and 0.9986, respectively; and those of Porous Structure PVC/CNT/SP10:4 are 2.435 kPa^−1^ and 0.9933, respectively. Among them, Porous Structure PVC/CNT/SP 10:3 achieves the optimal balance between sensitivity and linearity: its porosity not only provides sufficient deformation space to improve sensitivity but also maintains the stability of current response through more uniform pore distribution, thus ensuring better linearity. For Porous Structure 10:4, the higher content of white sugar results in higher porosity after dissolution, providing more deformation space for the material. When subjected to pressure, the reorganization effect of the CNT conductive network is more significant, and the number of conductive paths increases rapidly, leading to higher sensitivity. However, it should be noted that in the pressure range of 70–100 kPa, the sensitivity of the PVC/CNT/SP10:4 group shows an abnormal increase to a certain extent. This is because higher porosity increases the randomness of pore distribution, causing the material deformation to be more prone to nonlinear characteristics when pressure changes. This further reduces the regularity of current changes, which is not conducive to expanding the linear response range. Compared with the PVC/CNT/SP10:4 group, the porosity of the PVC/CNT/SP10:3 group matches the proportion of the PVC matrix more reasonably: the material is less likely to undergo plastic deformation or structural fatigue after repeated pressurization, resulting in higher long-term reliability. Therefore, Porous Structure 10:3 was selected for further optimization in subsequent experiments.

Based on the same 3 wt% CNT concentration, the performance comparison results between porous structure samples and non-porous-structure samples are shown in Figure 7b. It can be observed that the pressure sensors with porous structures exhibit better linearity in the 0–24 kPa pressure range. Meanwhile, their detection ranges are wider than those of sensors without porous PVC/CNT structures, and their sensitivities within the 0–24 kPa pressure range are also higher. This result indirectly confirms the effectiveness and feasibility of using white sugar as a sacrificial template for preparing porous structures.

### 3.3. Design of Gradientized Non-Uniform Porous Structures

To achieve a wider linear response range, inspired by the flexible pressure sensor with a bionic gradient porous structure based on carbon black/polydimethylsiloxane (CB/PDMS) prepared by Zhu et al. [20], this study proposes a gradient non-uniform porous structure design strategy. By adjusting the pore size, the sensor is dominated by high-density micropores in the surface layer to achieve a high-sensitivity response under low pressure, while the internal macroporous structure provides sufficient expanded deformation space under high pressure.

**Figure 8:**
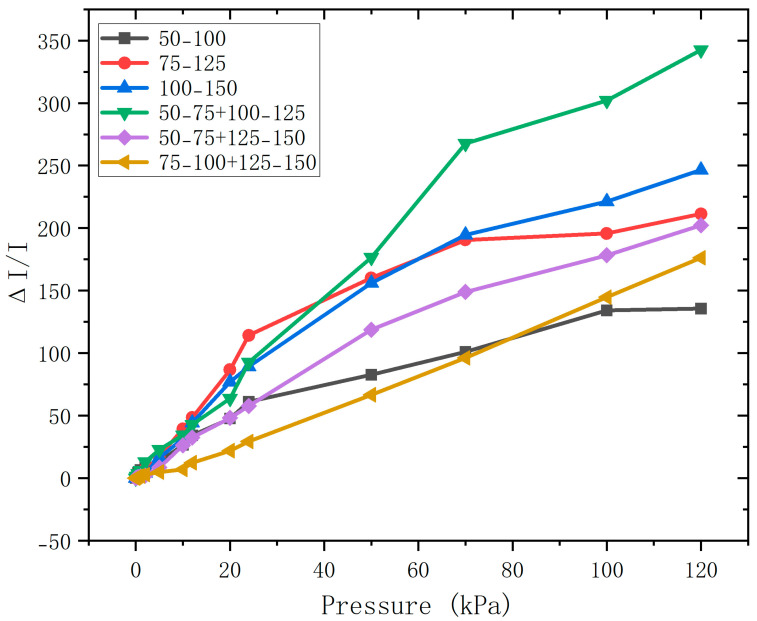
Plot of test results for all non-uniform porous samples.

The milled white sugar particles were sieved through screens of different mesh sizes to adjust their particle sizes, thereby regulating the pore sizes in the porous structure. Meanwhile, pores of different sizes were combined to form a non-uniform porous structure, ultimately resulting in a multi-gradient porous structure. Within the pore size range of 50–150 μm, white sugar templates of two gradient sizes were mixed at a 1:1 mass ratio, with a gradient difference of 25 μm. The test results are shown in Figure 8.

Table 1 presents a summary of the performance of all samples in Figure 8. As shown in the table, with the increase in the pore size of the porous structure, the sensor sensitivity shows a decreasing trend, while the linearity within the 0–120 kPa pressure range shows an increasing trend. For the PVC/CNT film with a thickness of 1000 μm, when a mixture of white sugar with pore sizes of 50–75 μm and 125–150 μm is used as the porous template, it exhibits the optimal sensitivity and linearity within the 120 kPa pressure range: the average sensitivity is 3.87 kPa^−1^, and the coefficient of determination (R^2^) is 0.9891. This sample is the optimal one, with a sensitivity of 3.87 kPa^−1^ and an R^2^ of 0.9891.

For the PVC/CNT film with a thickness of 1000 μm, within the pressure range of 0–120 kPa, whether for samples with smaller pore sizes (50–100 μm) or samples with better pore size continuity (75–125 μm, 100–150 μm), it can be observed that smaller pores contribute more significantly to sensitivity improvement. This is because during the pressure application process, larger pore structures are more likely to be squeezed and deformed, which helps alleviate stress concentration, while smaller pore structures can withstand higher pressure. As the pressure gradually increases, their deformation exhibits gradual compression, which maintains the continuity of conductive paths, avoids sudden resistance jumps, and thus enables high-sensitivity response over a wide pressure range. Selecting a pore size combination with appropriate dispersibility and gradient difference (50–75 μm + 125–150 μm) can achieve the synergistic effect of small and large pore sizes. This allows the fabricated porous structure pressure sensor to not only alleviate stress concentration but also maintain high sensitivity within a wider pressure-sensing range, ultimately achieving a wide linear sensing range of 0–120 kPa.

### 3.4. Characterization and Analysis of Gradient Porous Structures with Broad-Linearity Mechanisms

This study combines scanning electron microscopy (SEM), in situ electrical resistance testing, and dynamic cyclic loading experiments to reveal the stepwise deformation mechanism of gradient porous structures under pressure and the evolution law of their conductive networks. The results show that the gradual collapse characteristic of gradient pores can effectively alleviate the stress concentration problem prone to occur in traditional uniform porous structures, thereby achieving a linear current–pressure correlation over a wide pressure range.

**Figure 9:**
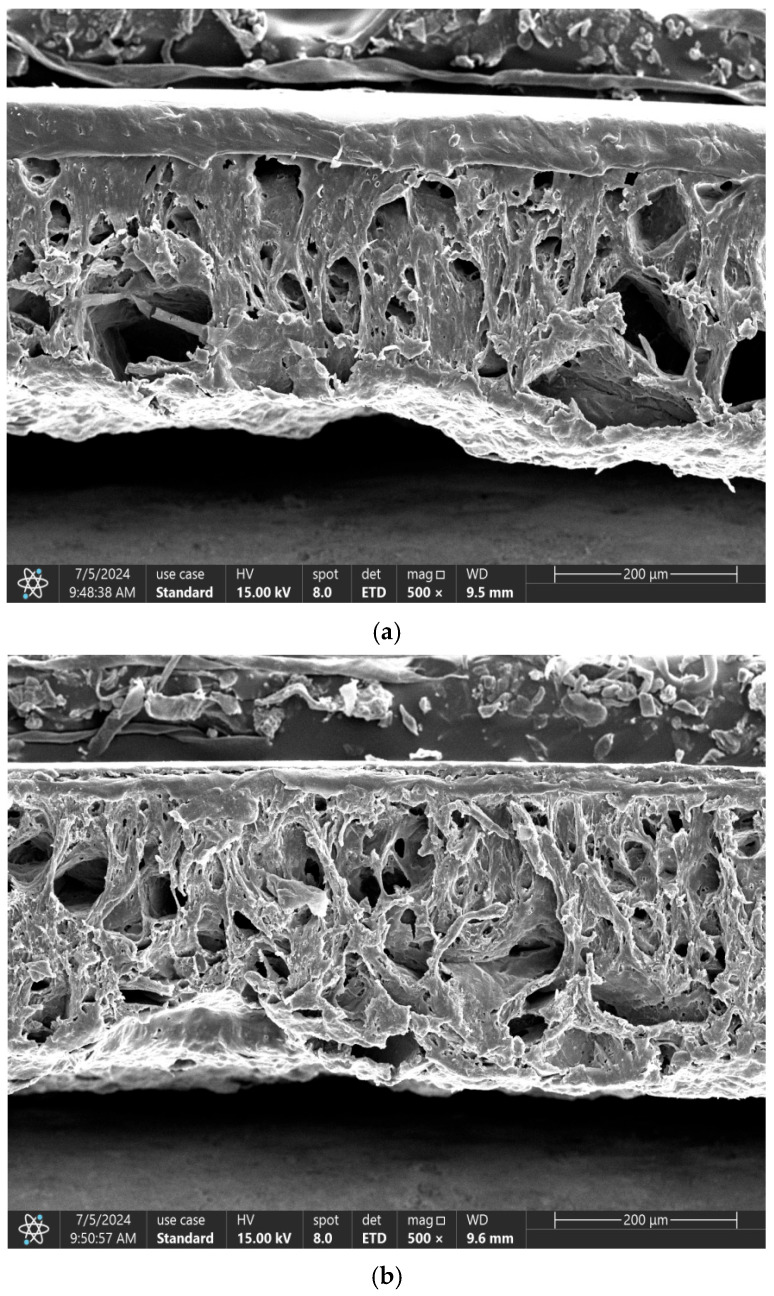

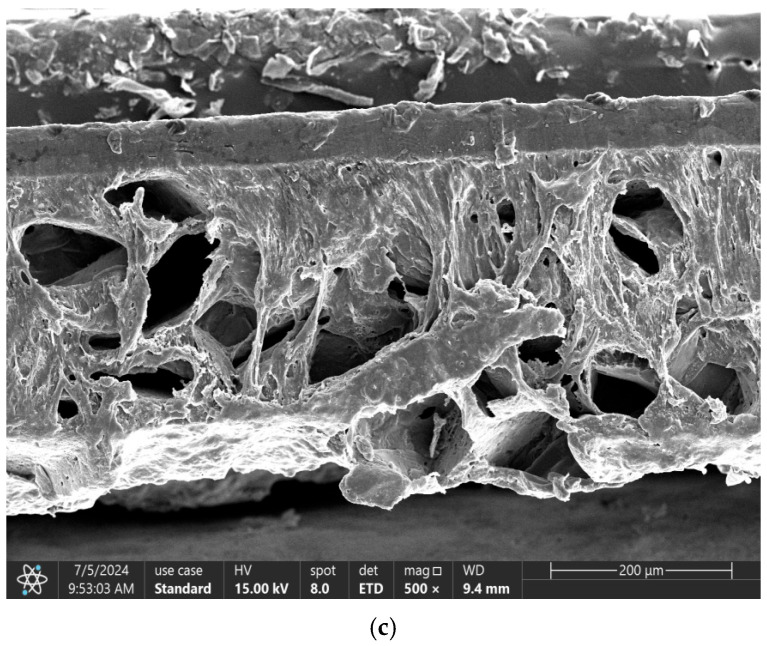
Cross-sectional and enlarged SEM images of sensors with three different non-uniform porous structures ((**a**) grouping of 50–75 um + 125–150 um; (**b**) grouping of 50–100 um; (**c**) grouping of 100–150 um).

The main resistance changes at each compression stage are highlighted in Figure 9. By observing the cross-sections of different non-uniform porous structures, it can be seen that the 50–75 μm + 125–150 μm group contains both large and small pore structures; the 50–100 μm group is dominated by smaller pores; and the 100–150 μm group is dominated by larger pores. Among these groups, the 50–75 μm + 125–150 μm group exhibits the optimal linearity.

**Figure 10:**
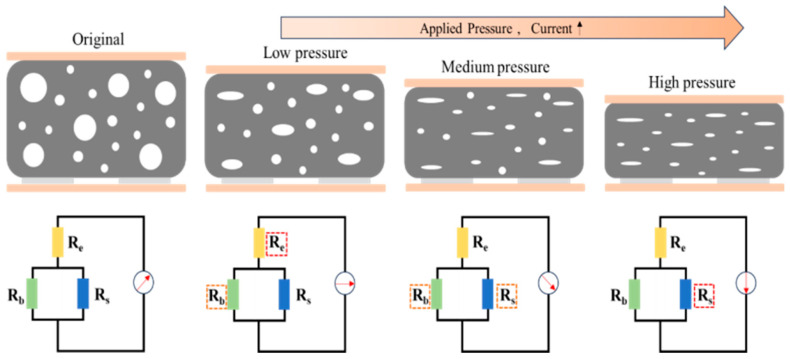
Schematic of the sensing mechanism of a wide linear flexible pressure sensor.

The sensing mechanism of the non-uniform porous structure sensor is illustrated in Figure 10. In this study, the applied pressure alters the contact state between three key components (i.e., interdigital electrodes, macroporous structures, and microporous structures). On the one hand, the porous sensing layer deforms under applied pressure, increasing the contact area between the interdigital electrodes and the porous sensing layer. On the other hand, the number of contact points between macroporous structures and between microporous structures increases. These changes collectively improve the continuity of conductive pathways, thereby enabling a sensitive response to pressure.

The optimized conductive pathway described above reduces resistance and increases current, thereby converting pressure input into current output. Theoretically, the total resistance Rt of the sensor can be expressed as Equation (1), where Re is the contact resistance between the sensing layer and the electrode layer; Rb is the resistance generated by the additional formation of carbon nanotube (CNT) contacts between macroporous structures under pressure; Rs is the resistance generated by the additional formation of CNT contacts between microporous structures under pressure.

(1) $$R_{t}=R_{e}+\frac{1}{\frac{1}{R_{b}}+\frac{1}{R_{s}}}$$

When pressure is applied to the sensor, during the low-pressure stage, the contact area between the sensing layer and the electrode layer increases, causing the contact resistance Re to decrease rapidly. Due to their loose structure, macroporous structures preferentially undergo elastic deformation under low pressure: their pore walls gradually bend or come into contact with each other, leading to a gradual reduction in pore volume and even localized collapse. This process significantly increases the number of contact points between the conductive filler (carbon nanotubes, CNTs), forming new conductive pathways and thereby causing the macropore contact resistance *R_b_* to start decreasing gradually. In contrast, microporous structures hardly deform under low pressure due to their denser structure or thicker pore walls, so there is no significant change in the micropore contact resistance *Rs*. At this stage, micropores mainly act as rigid supports to maintain the stability of the material’s overall structure and prevent mechanical failure caused by excessive collapse of macropores.

In the medium-pressure stage, macroporous structures are further compressed; some macropores collapse completely and enter the densification stage, at which point the conductive paths tend to saturate. During this process, the contribution of macropores to resistance change gradually weakens, and their contact resistance Rb decreases slowly to a minimum value. At the same time, microporous structures begin to participate in deformation: due to their high elastic modulus, micropores can only be compressed under higher pressure, and this compression mainly occurs in local stress concentration regions. As pressure continues to be applied, the micropore walls gradually bend or come into contact with each other, promoting the rearrangement of the carbon nanotube (CNT) network and the reconstruction of conductive paths, which in turn leads to a decrease in the micropore contact resistance Rs.

In the high-pressure stage, macroporous structures have completely collapsed or densified and can no longer be compressed further. As the number of conductive paths stabilizes, the change in the macropore contact resistance Rb almost ceases. During this stage, microporous structures continue to be compressed under high pressure, with their pore walls coming into close contact and even undergoing plastic deformation. At this point, the spacing between carbon nanotubes (CNTs) inside most micropores approaches the threshold of the quantum tunneling effect, which further optimizes the conductive paths. Consequently, the micropore contact resistance Rs decreases rapidly to a minimum value, thereby maintaining the sensor’s sensitivity in the high-pressure stage.

In summary, this study adopts a non-uniform porous structure design, whose core advantage lies in the fact that pores of different sizes can be gradually closed within different pressure ranges. This characteristic enables the resistance to change progressively with increasing pressure, rather than being concentrated in a specific pressure interval. This progressive closure process not only broadens the sensor’s operating range, but also makes the relationship between current and pressure changes more linear and smooth, effectively avoiding sudden current changes or premature saturation, and ultimately significantly improving the sensor’s linearity.

## 4. Results

### 4.1. Sensitivity

**Figure 11:**
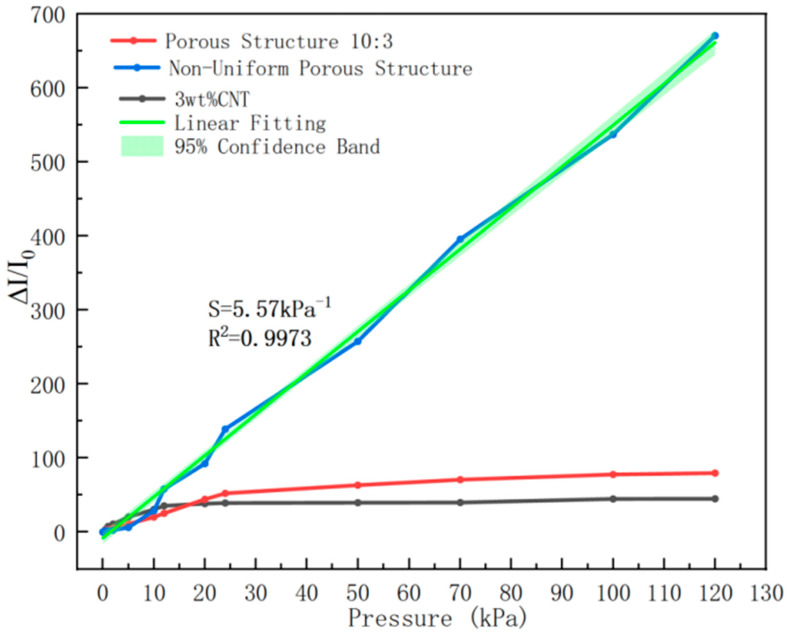
Wide-linearity flexible pressure sensor sensitivity and linearity.

Sensitivity is a core indicator for measuring a sensor’s ability to respond to pressure changes. As shown in Figure 11, within the wide pressure range of 0–120 kPa, the relative current change (ΔI/I_0_) of the sensor exhibits a highly linear relationship with the applied pressure, with a coefficient of determination R^2^ > 0.99 and a sensitivity of 5.57 kPa^−1^. Notably, the sensitivity improvement is particularly significant in the low-pressure region (0–20 kPa), which is attributed to the rapid deformation of micropores in the gradient porous structure, enabling gradual reconstruction of the conductive network. In the high-pressure region (20–120 kPa), the gradual compression of macroporous structures can effectively maintain the continuity of conductive pathways, thereby ensuring the stability of sensitivity. In addition, by comparing samples with ordinary porous structures and non-porous structures, it can be concluded that the pressure sensor with the optimized non-uniform porous structure designed in this study exhibits significant improvements in both sensitivity and linearity within the range of 0–120 kPa. This result indicates that the multi-gradient non-uniform porous design has successfully achieved the synergistic optimization of high sensitivity and linear response over the wide pressure range of 0–120 kPa.

### 4.2. The Pressure Gradient Test

**Figure 12:**
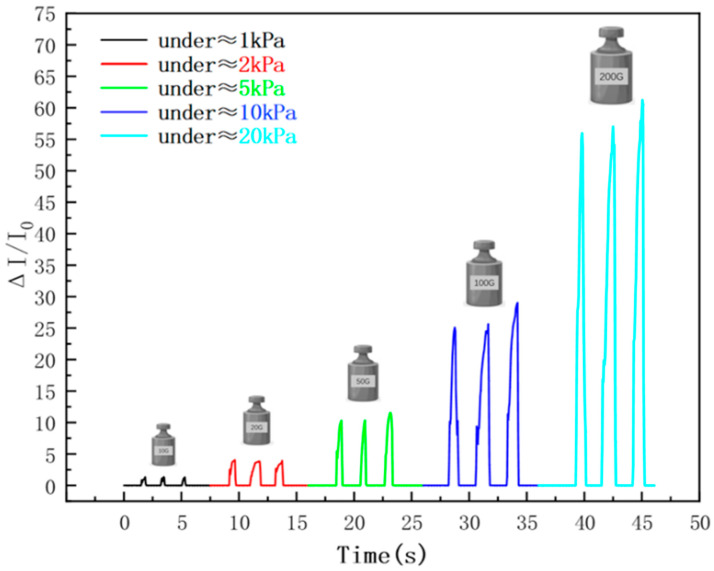
Pressure gradient response curve of wide-linearity flexible pressure sensor in the range of 1–20 kPa.

To verify the sensor’s dynamic discrimination capability for pressure changes, this study conducted three loading–unloading cycle tests at pressures of approximately 1 kPa, 2 kPa, 5 kPa, 10 kPa, and 20 kPa, respectively. Figure 12 shows the sensor’s response curves when stepwise incremental pressures are applied within the range of 0–20 kPa. The results indicate that the sensor can achieve stable and accurate feedback under different pressures, clearly distinguish step pressure changes, and exhibit a linear growth trend of output current with increasing pressure. Additionally, the signal fluctuation rate between each pressure step is less than 5%. Further analysis reveals that the sensor’s conductive network does not saturate or fracture within the test pressure range. This phenomenon verifies the significant advantage of the multi-gradient non-uniform porous structure in dispersing stress concentration.

### 4.3. The Response Time and Recovery Time

**Figure 13:**
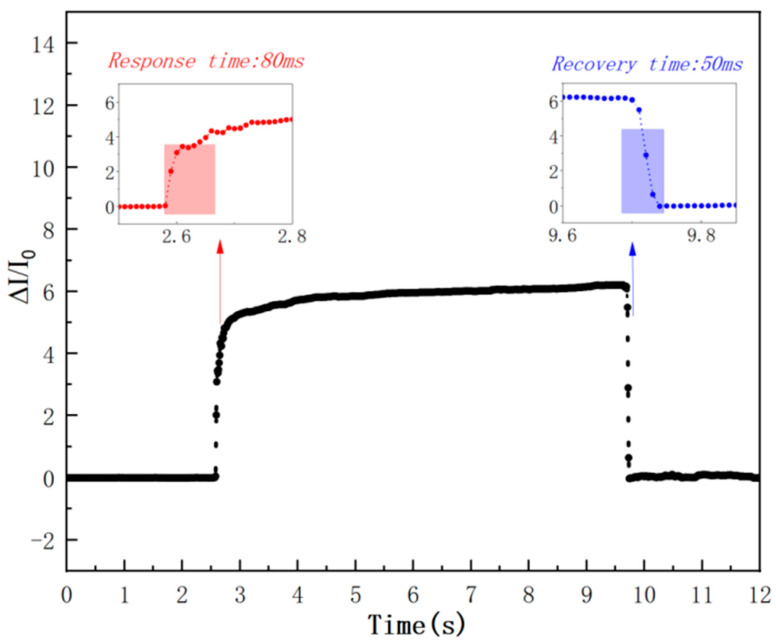
Response time and recovery time of a wide-linearity flexible pressure sensor.

The dynamic response characteristic is a key parameter for evaluating a sensor’s real-time monitoring capability, and it is usually characterized by the sensor’s response time to pressure and recovery time. A pressure load of 5 kPa was applied to the sensor surface, and after maintaining this constant load for approximately 7 s, the pressure stimulus was quickly removed. The test results are shown in Figure 13: the response time (under pressure application) and recovery time (under pressure removal) of the sensor are approximately 80 ms and 50 ms, respectively, reaching a millisecond-level dynamic response. The sensor’s fast response is attributed to the elastic deformation characteristics of the gradient porous structure and the high electrical conductivity of the carbon nanotube (CNT) network; the slight lag in response time, however, is related to the viscoelastic relaxation effect of the polymer matrix (PVC).

Figure 13: Dynamic response and recovery characteristics of the wide-linearity flexible pressure sensor under a 5 kPa pressure load. The main curve shows the normalized current change (ΔI/I_0_) over time. The red arrow and inset highlight the response time (80 ms), defined as the time required to reach 90% of the maximum signal after pressure application. The blue arrow and inset show the recovery time (50 ms), representing the time to drop to 10% of the peak signal after pressure release. The black dots indicate discrete data points collected during the transient process, demonstrating the high temporal resolution of the sensor. The insets provide magnified views of the rising and falling edges for clarity.

### 4.4. Cyclic Stability

**Figure 14:**
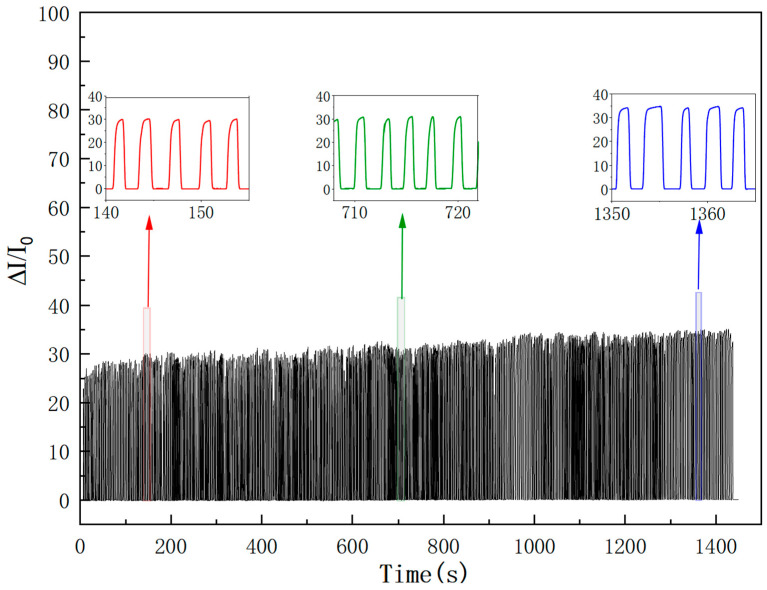
Cyclic stability of wide-linearity flexible pressure sensors.

Long-term cyclic stability is a crucial guarantee for sensors to achieve practical application. In this study, the fabricated sensor was subjected to long-term cyclic testing under a pressure of 10 kPa, and the results are shown in Figure 14. The figure presents the amplified local response curves of the initial stage, mid-term stage, and final stage throughout the entire testing process. It can be observed that the sensor exhibits minimal baseline drift, and no significant changes in the response curves occur during the entire cyclic pressure testing process. The slight fluctuations in the curves are attributed to the combined effects of the sensor’s own structural properties and external testing conditions. From a material perspective, long-term cyclic loading may induce microplastic deformation in the elastic matrix. From a testing system perspective, differences in the dynamic load adaptability of the testing platform can introduce systematic errors, which mainly originate from the mechanical hysteresis effect during the pressure loading and unloading processes.

Taken together, the fabricated sensor still maintained excellent stability and durability after 500 cyclic load tests under a pressure of 10 kPa. This performance advantage is attributed to the strong interfacial bonding between the conductive filler (carbon nanotubes, CNTs) and the PVC matrix, as well as the inherent anti-fatigue properties of the gradient porous structure itself. Further verification via microscopic scanning electron microscopy (SEM) analysis confirmed that no significant collapse or fracture of the porous structure occurred after cyclic testing, directly validating the structural durability of the sensor.

Figure 14: Cyclic stability of the wide-linearity flexible pressure sensor under a constant pressure of 10 kPa for 500 loading-unloading cycles. The main curve shows the normalized current change (ΔI/I_0_) over time, demonstrating minimal baseline drift and consistent signal amplitude throughout the test. Three inset plots highlight the amplified local response curves at different stages:

Red: initial stage (t = 140–150 s), showing the early response behavior;

Green: mid-term stage (t = 710–720 s), indicating stable performance during prolonged operation;

Blue: final stage (t = 1350–1360 s), confirming no significant degradation after 500 cycles.

The black line represents the continuous output signal, while the arrows indicate the corresponding regions in the main curve. The results demonstrate excellent long-term durability and reliability.

### 4.5. Application

This sensor shows promising application potential in cutting-edge fields such as human motion and health monitoring.

**Figure 15:**
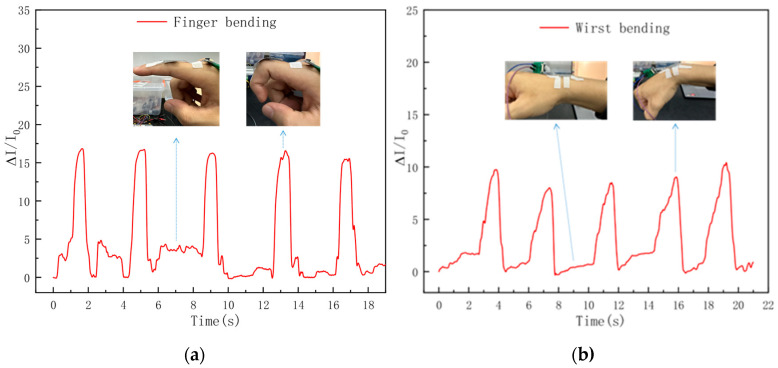
Sensor real-time response for (**a**) finger bending and (**b**) wrist bending detection.

The flexible pressure sensors fabricated in this study were affixed to the right index finger joint and wrist joint of volunteers, respectively, to detect the current response of the sensors during the joint flexion of the volunteers. As shown in Figure 15a, when the finger joint was in an extended state at the initial stage, there was no change in the relative current. In contrast, during the process of repeated inward flexion of the finger joint five times in a short period, the relative current of the sensor exhibited five corresponding responses. This is because when the finger joint bends, the attached sensor is compressed and deformed, resulting in a certain degree of closure of its internal porous structure. The increase in conductive pathways consequently leads to a rise in the output current. As shown in Figure 15b, it can be observed that the peak value of the relative current response of the wrist joint is lower than that of the finger joint. This is attributed to the fact that the flexion amplitude of the human finger joint is larger, which causes a greater compression degree on the sensor, thus resulting in a higher peak value of the relative current induced by the finger joint flexion.

The above results demonstrate that the fabricated flexible pressure sensor can realize the detection of human joint movements. Meanwhile, it is feasible to distinguish the movements of different joints by the magnitude of the relative current response of the sensor, which also indicates the great application potential of the sensor in the fields of wearable electronic devices and motion detection.

**Figure 16:**
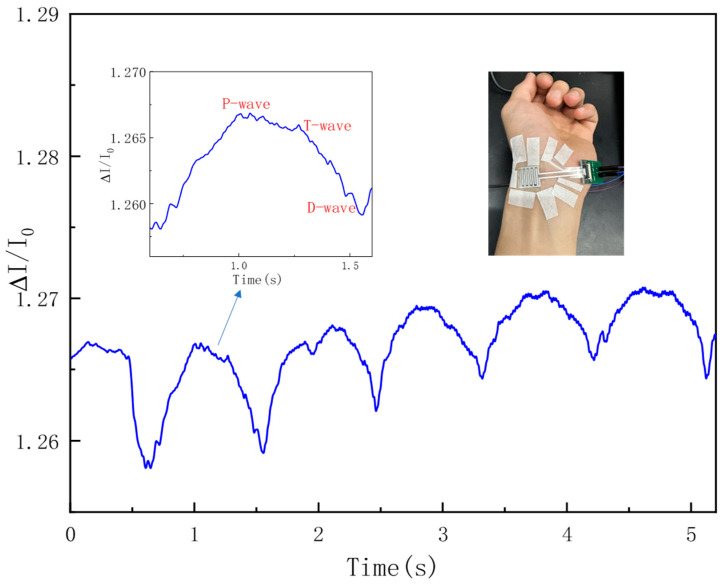
Real-time radial pulse waveform showing P-, T-, and D-waves.

To verify the feasibility of detecting human pulse signals, the prepared flexible piezoresistive pressure sensor was tightly affixed and secured to the left radial artery of a volunteer using adhesive tape for real-time pulse monitoring. As shown in Figure 16, the sensor was able to identify the volunteer’s pulse. When a segment of the pulse signal was amplified, three distinguishable characteristic peaks could be observed, namely the percussion wave (P), tidal wave (T), and diastolic wave (D).

This indicates that the sensor fabricated in this study features high sensitivity and a low detection limit; it can accurately detect minor pressure changes in various parts of the human body and is capable of measuring human pulse signals. Meanwhile, this also demonstrates the sensor’s potential application value in the fields of health monitoring and disease diagnosis.

Figure 16: Real-time radial pulse waveform recorded by the flexible piezoresistive pressure sensor, showing the characteristic P-, T-, and D-waves. The blue line represents the raw pulse signal, with a magnified segment (inset) highlighting the key features. The blue arrow points to the corresponding region in the full waveform. The red labels indicate the percussion wave (P), tidal wave (T), and diastolic wave (D), respectively.

## 5. Conclusions

Flexible piezoresistive pressure sensors are in increasing demand in the fields of wearable health monitoring and intelligent interaction; however, existing technologies struggle to synergistically optimize the core trade-off between high sensitivity and a wide linear range. This study proposes a gradient-based non-uniform porous structure design concept. Through the synergistic deformation mechanism of large and small pores—where large pores preferentially collapse to achieve a highly sensitive response in the low-pressure region, and small pores gradually compress to delay the saturation of the conductive network in the high-pressure region—a new approach is provided to address the trade-off challenge between sensitivity (S) and linear range (LR).

Based on the above design, this study prepared the sensing layer using PVC/CNT composite paste and a bimodal template of white sugar (a mixture of 50–75 μm and 125–150 μm particles), and optimized the key process parameters via orthogonal experiments. The optimal parameters were finally determined as follows: CNT concentration of 3 wt% and porosity ratio (PVC/CNT/SP) of 10:3. The resulting sensor exhibited excellent performance within the pressure range of 0–120 kPa: a high linearity with R^2^ > 0.99, a sensitivity of 5.57 kPa^−1^, a millisecond-level dynamic response (response time 80 ms/recovery time 50 ms), and cyclic stability exceeding 500 cycles. This result fully verifies the effectiveness of the synergistic mechanism of non-uniform pores.

The performance of this sensor was compared with some existing sensors in the literature for pressure monitoring, as shown in Table 2. Compared with other existing works in references [1,2,3], the sensor proposed in this paper provides a wide pressure linear range (120 kPa) and higher sensitivity (5.57 kPa^−1^).

This sensor shows promising application potential in cutting-edge fields such as human motion monitoring and electronic skin; however, the large-scale production capability of the current template-based fabrication process and the long-term environmental stability of the sensor itself still need improvement. Future research can be carried out in two aspects—first, exploring precise pore regulation technologies to further optimize the structure–performance matching relationship; and second, integrating machine learning algorithms to enhance signal decoupling capabilities—thereby expanding the sensor’s practical application scope in complex dynamic scenarios.

## Figures and Tables

**Table 1:** Summary plot of sample properties for all non-uniform porous-structured PVC/CNT films.

Porous Structure Type (μm)	Average Sensitivity (0–120 kPa)	Average R^2^ (0–120 kPa)
PVC/CNT/SP50–100 um	2.27 kPa^−1^	0.9247
PVC/CNT/SP75–125 um	1.80 kPa^−1^	0.9276
PVC/CNT/SP100–150 um	1.76 kPa^−1^	0.9712
PVC/CNT/SP50–75 um + 100–125 um	2.22 kPa^−1^	0.9814
PVC/CNT/SP50–75 um + 125–150 um	3.87 kPa^−1^	0.9891
PVC/CNT/SP75–100 um + 125–150 um	1.41 kPa^−1^	0.9653

**Table 2:** Comparison between the proposed sensor and the existing literature.

Pressure Linear Range	Sensitivity	R^2^	Ref
0–25 kPa	1.2 kPa^−1^	>0.99	Ref. [23]
0–100 kPa	0.045 kPa^−1^	>0.99	Ref. [24]
0–17 kPa	9.38 kPa^−1^	>0.99	Ref. [25]
0–120 kPa	5.57 kPa^−1^	0.9973	This work

## Data Availability

The data presented in this study are available on request from the corresponding author.
